# Sperm-Guiding Unconventional Prostaglandins in *C. elegans*: Synthesis and Signaling

**DOI:** 10.3390/metabo11120853

**Published:** 2021-12-08

**Authors:** Ekta Tiwary, Muhan Hu, Jeevan K. Prasain

**Affiliations:** 1 Department of Medicines, University of Alabama at Birmingham, Birmingham, AL 35205, USA; etiwary@uab.edu; 2 Medical Scientist Training Program, University of Alabama at Birmingham, Birmingham, AL 35205, USA; mhu1@uab.edu; 3 Department of Pharmacology and Toxicology, University of Alabama at Birmingham, Birmingham, AL 35294, USA

**Keywords:** sperm guidance, prostaglandin, *C. elegans*, TGF-β, Cox-independent pathway

## Abstract

Prostaglandins comprise a family of lipid signaling molecules derived from polyunsaturated fatty acids and are involved in a wide array of biological processes, including fertilization. Prostaglandin-endoperoxide synthase (a.k.a. cyclooxygenase or Cox) initiates prostaglandin synthesis from 20-carbon polyunsaturated fatty acids, such as arachidonic acid. Oocytes of *Caenorhabditis elegans* (*C. elegans*) have been shown to secrete sperm-guidance cues prostaglandins, independent of Cox enzymes. Both prostaglandin synthesis and signal transduction in *C. elegans* are environmentally modulated pathways that regulate sperm guidance to the fertilization site. Environmental factors such as food triggers insulin and TGF-β secretion and their levels regulate tissue-specific prostaglandin synthesis in *C. elegans*. This novel PG pathway is abundant in mouse and human ovarian follicular fluid, where their functions, mechanism of synthesis and pathways remain to be established. Given the importance of prostaglandins in reproductive processes, a better understanding of how diets and other environmental factors influence their synthesis and function may lead to new strategies towards improving fertility in mammals.

## 1. Introduction

Prostaglandins (PGs) are a family of important lipid signaling molecules produced in most tissues and organs from polyunsaturated fatty acids (PUFAs) such as arachidonic acid (AA) (C20:4) by Cox enzymes. They are implicated in regulating human reproduction, inflammation, neurological function, and cancer progression, and act as short-lived, local hormones. PG research started more than 80 years ago when the American gynecologists Kurzrok and Lieb first discovered that a factor in human semen could promote uterine contractions. These observations were confirmed by Godblatt (1933) and von Euler (1936) that a group of substances with smooth muscle stimulating and vaso-depressive properties exist in human semen, prostate and seminal vesicles [1,2]. von Euler believed that these substances were produced in the prostate gland and therefore, named them “prostaglandins”. The structures of some of the PGs were first identified in 1962 by Swedish biochemist-physician Bergström [3]. Later, research demonstrated that PGs are inflammatory mediators and aspirin-like drugs have analgesic effects owing to inhibition of PG synthesis [4,5,6].

PG-like compounds have been reported in primitive insect *Thermobia domestica* through enzymatic action of lipoxygenase [7]. Another example is the Caribbean coral *Plexaura homomalla* which produces highest levels PGs with a unique 15R stereospecificity [8]. PGs also have been found in pathogenic yeasts and may play roles in pathogen host interactions [9]. While PGs have been identified in many animals, including invertebrates, their genetic and underlying mechanism of action are still unclear.

Although Cox enzymes mediate the canonical PG synthesis pathway, non-enzymatic mechanisms, can also generate PGs and PG-like compounds [10,11]. The isoprostanes are a unique series of PG-like compounds formed in vivo from free radical initiated lipid peroxidation of AA under oxidative stress conditions [10]. Over the past 10 years, we have shown that PGs in *C. elegans* can be synthesized by an unconventional Cox-independent mechanism and are dynamically regulated by pheromones and nutritional cues [12,13,14].

The importance of PGs to human health become evident when it was found that their levels are significantly altered by various physiological conditions [15,16,17,18]. For example, fever a hallmark of infection and inflammation is mediated by PGE2 [19,20]. It has also been reported that level of PGE2 plays a pivotal role in reproduction [2,21]. However, it is still not clear how PGs that are synthesized by different pathways are involved in diverse biological processes. This review article summarizes the different pathways of PG synthesis and their roles in reproductive processes with special emphasis on Cox-independent PGs and signaling mechanisms that influence their production and reproductive functions.

## 2. Prostaglandin Synthesis

### 2.1. Cox-Mediated PG Synthesis

**Figure 1:**
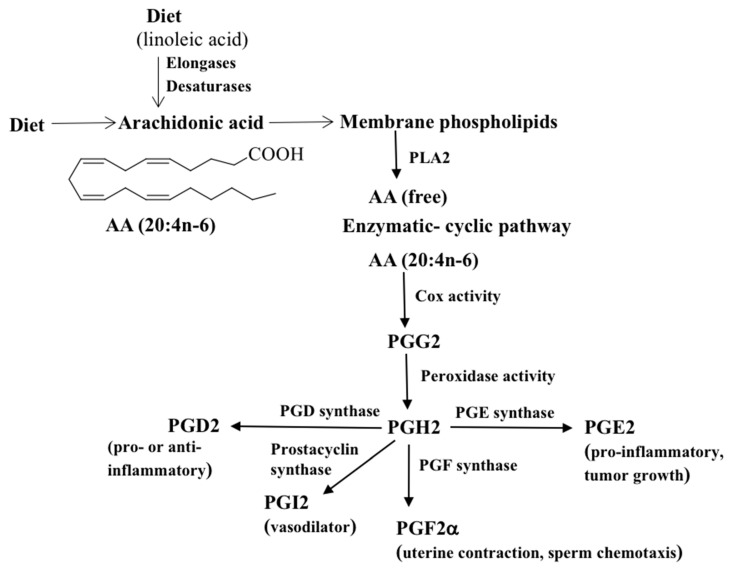
A general overview of the Cox-mediated PG synthesis and their major functions. Mammals, being incapable of synthesizing PUFAs such as linoleic or arachidonic acid, they must be provided in the diet. AA liberated from cellular membrane phospholipids such as phosphatidylocholine by the action of phospholipase A2 (PLA2) is converted to PGH2 via PGG2 by Cox-1/Cox-2 enzymes. PGH2 acts as a substrate for PG synthases to give rise to different PGs.

The conventional wisdom is that Cox enzymes are sole enzymes responsible for initiating the synthesis of PGs. The Cox enzyme was first purified from sheep seminal vesicles in the 1970s [22,23]. There are at least two Cox isoforms, Cox-1 and Cox-2, and both catalyze the formation of PGs [24]. The Cox-1 enzyme is constitutively expressed in the gastrointestinal tract and is responsible for maintaining the mucosa of the stomach and intestine [25,26]. Cox-2 is an inducible enzyme, predominantly produced in response to inflammatory stimuli at the site of inflammation [27,28]. Cox converts AA into the bicyclic endoperoxide PGG2, which is then reduced to PGH2. The PGH2 intermediate is further converted into bioactive forms PGs by specific PGD, PGE, and PGF synthases (Figure 1) [29,30]. For example, the prostaglandin D synthase converts PGH2 into PGD2. Likewise, PGE synthase transforms PGH2 to PGE2 and dehydration of PGE2 yields PGA2, which, on migration of the cyclopentene double bond, affords two other cyclopentenone PGs, first PGC2 and then PGB2. PGI2 (prostacyclin), being chemically unstable, is hydrolyzed to stable 6-keto PGF1α. PGF2α can be formed by various mechanisms. (I) The 9-keto group of PGE2 can be reduced to PGF2, (II) the 11-keto group of PGD2 can be reduced to PGF2, and (III) the 9,11-endoperoxide group of PGH2 can be reduced to PGF2. Enzymes belonging to the aldo-keto reductase (AKR) family catalyze the conversion of PGD2 and PGE2 to PGF2. Specific PGs are denoted by a letter A-H, representing oxygen substitution on the cyclopentane ring structure, and by a number, representing the number of cis double bonds in the lipid [31,32]. For example, F- series PGs such as PGF2α are among the most abundant and ubiquitous PGs, and they contain two hydroxyl groups in the cyclopentane ring and two double bonds in the side chains. A general overview of Cox-mediated PG synthesis and their functions is shown in Figure 1. PGs exert their autocrine or paracrine function via specific G protein-coupled receptors [33]. In addition, selected PGs are weak agonists for the aryl hydrocarbon receptor and mediate the biological actions of many environmental toxins [34].

### 2.2. Non-Enzymatic Pathways

A broad spectrum of PG-like compounds is produced non-enzymatically by free-radical initiated oxidation of PUFAs. Morrow and Roberts in 1990 discovered these compounds as F2-isoprostanes (IsoPs) [35,36]. In this pathway, a complicated mixture of 64 isomers can be formed comprising four regioisomeric families (5-, 12-, 8-, or 15-series) each with 8 racemic diastereomers [37,38,39]. IsoPs are structurally distinct from PGs with regard to the orientation of side chains, that are predominantly oriented ***cis*** to the prostane ring in IsoPs [10]. Another point of difference between PG and IsoP synthesis is that IsoPs are initially formed in situ in phospholipids and subsequently released by phospholipase A2 whereas PUFAs are released first from membrane phospholipids via receptor/G-protein-initiated activation of phospholipase A2 in PG synthesis [40]. IsoPs are chemically stable and can be detected in biological samples (fluids and tissues) [11]. Among IsoPs, 8-isoPGF2α (a diastereoisomer of PGF2α) is regarded as a marker of oxidative stress [41]. However, studies have also shown that increased levels of this compound do not necessarily correspond to increased oxidative stress [42]. The formation of PGH2 synthase mediated 8-IsoPG has also been reported in cultured human endothelial cells [43].

The Morrow and Roberts groups have indicated the generation of compounds identical to those Cox-derived PGE2 and PGD2 from the peroxidation of AA, indicating the possibility that a second pathway exists for the formation of bioactive PGs in vivo that is independent of Cox [44].

### 2.3. Unconventional Cox-Independent Pathway

Although the *C. elegans* genome does not encode Cox homologs, we have shown that the *C. elegans* oocytes synthesize F-series PGs from arachidonic acid [45]. It also produces a wide range of proteins similar to those involved in human PG metabolism such as PG synthases, thromboxane synthases, cytochrome P450s, and phospholipase. Moreover, the evolutionary ancestors of Cox enzymes, the myeloperoxidases, are not required for PG synthesis [12]. This novel PG synthesis pathway is also active in mice and found in human follicular fluids (HFF) [14,46]. Over 50% of PGF2**α** isomers are formed independent of Cox in Cox1/Cox2 double KO mice [14]. HFF contains PGs synthesized by both Cox-dependent and Cox-independent pathway(s). While Cox-independent PGs are abundant in HFF, their functions and regulatory pathways are unknown. Since reduced PG levels dramatically impair fertility due to sperm loss from the oviduct, Cox-independent PGs have an important function in *C. elegans* fertilization. These PGs may influence sperm motility and thus fertilization event in humans as well. For example, PGF1α binds with high affinity to the calcium channel of sperm (CatSper), in human sperm important for motility [47,48].

There are a number of important features of Cox-independent PG synthesis pathway(s). First, Cox-independent PGs are formed with a signature profile through a biologically regulated mechanism, rather than ROS-mediated free radical oxidation [45]. The most hydrophilic stereoisomer co-elutes with 8-isoPGF2α, followed by 5iPF2VI, and PGF2α based on their retention times and MS/MS comparison with standards in LC-MS/MS. The fourth peak does not co-elute with available standards.

This pathway of PG synthesis is not significantly affected by specific anti-oxidants, Cox-, Lox-, and Cyp- inhibitors, suggesting that these PGs are formed through a novel, biologically regulated mechanism in *C. elegans* [45]. Unlike Cox-derived PGs, no detectable 6-keto PGF1α is produced in this pathway. Another characteristic of this pathway is that 8-isoPGF2α and 5iPF2VI are formed enzymatically as opposed to ROS-mediated synthesis [38]. This pathway is tissue specific and in adult worms, PGs are predominantly generated in the oogonia (oocytes and their precursors). Further, it is dynamically regulated by pheromones and nutritional cues in the external environment [13,14]. Since PGs are implicated in many pathophysiological processes in the human body, the unconventional PG synthesis pathway may have significant clinical implications [46].

## 3. PUFAs, PGs and Reproduction

### 3.1. PUFAs

PUFAs contain at least two double bonds in their carbon backbone separated by a methylene group. Desaturase enzymes insert double bonds into the carbon chain, while the elongation system increases the length of the chain. Mammals lack the desaturase enzymes necessary to convert monounsaturated fatty acids such as oleic acid (18:1) into PUFAs. Linoleic (18:2n-6) and linolenic acids (18:3n-3) are called essential fatty acids and must be provided by dietary source. Plants can synthesize PUFAs de novo, and therefore, are important dietary sources. *C. elegans* synthesizes a wide variety of fatty acids using ∆12, 3, 5, 6, and ∆9 desaturases [49,50,51].

Mammals are capable of desaturating and elongating linoleic and linolenic acids to produce AA (20:4n-6) eicosapentaenoic acid (EPA, 20:5n-3), respectively. AA and EPA are precursors of the eicosanoids (oxygenated metabolites derived from C-20 PUFAs, including PGs). They are transported from the intestine to oocytes yolk lipoprotein complexes and RME-2 low-density lipoprotein (LDL) receptor mediates yolk endocytosis in *C. elegans* [52,53]. RME-2 loss causes sperm motility defects nearly identical to loss of PUFAs or oocytes, indicating that RME-2 is involved in PUFA transport and PG synthesis [54].

PGs are categorized into different series, based on the number of double bonds present in their structures. The series 1 PGs have one double bond produced from dihomo-γ-linolenic acid (DGLA). The Series 2 and 3 PGs contain two and three double bonds produced from AA and EPA, respectively. We have shown that these PUFAs are the precursors for sperm-guiding PG formation and converted into more than 10 structurally related F-series PGs in *C. elegans*, which function collectively and largely redundantly to guide sperm to the fertilization site [13].

We also discovered that F-series PGs are significantly enriched in extracts from wild-type adults compared to mutants lacking germ cells *glp-4(bn2)* based on LC-MS/MS analysis. About 75% of PG was reduced in *glp-4* mutants indicating that germ line is required for PG synthesis in *C. elegans* [13]. Taken together, these results indicate that different series of PGs are formed from omega-3 and-6 PUFAs and their levels in a diet may influence reproductive output. However, a more complete understanding of transport of these PUFAs to ovarian cells and their metabolism is still lacking.

### 3.2. PG and Reproduction

It is becoming increasingly clear that PGs influence multiple reproductive processes, including ovulation and fertilization. Several studies have indicated that PG inhibitors such as non-steroidal anti-inflammatory drugs (NSAIDs) are associated with reversible female infertility, and ovulation disorders [55]. For example, naproxen sodium, diclofenac, and piroxicam have been linked to infertility in women receiving treatment for inflammatory joint diseases, suggesting NSAID consumption may have adverse effects for ovulation and pregnancy.

Genetic studies of mouse Cox genes have provided significant insight into PG reproductive functions. Cox-1 deficient female mice are fertile, but delayed parturition results in neonatal death [56]. Cox-2 knockout mice, on the other hand, are infertile due to defects in ovulation, fertilization, implantation, and decidualization [57]. Cox-1 and Cox-2 double null mutants die shortly after birth due to a failure of the ductus arteriosus to close. This defect is due to impaired PGE2 synthesis. In the Cox-dependent pathway, PGF2α is identified as the mammalian luteolytic hormone [58]. Cox-knockout mice have reduced levels of PGs and PG supplementation improves ovulation, fertilization, embryo development and early implantation [59]. These studies suggest that fertilization defects in Cox-2 mutant female mice may be associated with the possibility that PGs function to guide sperm to the oocyte.

Also supporting the role of PGs in ovulation and fertilization is that knockout of the PGE2 cell surface receptor EP2 causes limited expansion of the cumulus masses that surround oocytes [21]. Preovulatory gonadotropin surge causes a strong increase in Cox-2 expression in cumulus cells that persists in ovulated eggs. These cells express increased levels of EP2 receptor [60]. Female mice lacking EP2 have impaired ovulation resulting in a dramatic reduction in litter size when compared to control animals [61]. These results indicate that PGE2 is a key ovulatory mediator, although the underlying molecular mechanisms are not well-understood [62].

## 4. Sperm Guidance Cues

Fertilization is an important event for the formation of the embryo. A regulated sperm oocyte communication results in a successful fertilization. For this, sperm must meet the oocyte at right place and time to generate a viable embryo. Fertilization takes place either outside (external fertilization) or inside the female reproductive tract (internal fertilization).

Different guiding models that orient sperms towards eggs and improved techniques for evaluation of sperm motility have been developed. These models included chemotaxis, rheotaxis, contractile forces, and thermotaxis [63,64,65,66]. Chemotaxis refers movement of a motile cell or organism in a concentration gradient of an external chemical factors, i.e., chemoattractants [67,68]. Rheotaxis is the mechanism where sperm swims against or same direction of the fluid flow in the oviduct. Cell secretion, muscle contraction and ciliary beating helps in the flow of oviduct fluid and as a result, oocyte and sperm cross their path. In vitro studies have shown that rheotaxis is a major taxic factor involved in mouse and human sperm taxis [64,69]. Another model is contractile forces, where contractions within the reproductive tract force sperm to move towards the oocyte as previously shown in *Drosophila* [65]. In thermotaxis, a directional movement of sperm has been reported to the temperature gradient in the reproductive tract [63].

Among these models, chemotaxis is the most studied model where the oocytes secrete sperm chemoattractants in the oviduct and help sperm to find the mature oocytes [13,54,70,71]. Chemistry of chemoattractants includes proteins, peptides and small molecules [72]. Small molecules such as sperm-activating and attracting factor (SAAF), dodeca-2,3-diynol and tryptophan are reported in studies of *Ciona intestinalis* (ascidian), *Montipora digitate* (coral) and *Haliotis rufescens* (abalone) respectively [73,74,75]. Aldehyde such as burgeonal and lyral are also identified as chemoattractants in mammals [76,77]. Additionally, small peptides (resact, Ser5 speract, asterosap, N-formylated peptide, atrial natriuretic peptide), proteins (alluring and RANTES, natriuretic peptide precursor A, and CRISP1), and lipids (progesterone and PGs) are also reported as chemoattractants in invertebrates and mammals [13,70,75,78,79,80,81,82,83,84,85,86,87].

In externally fertilized animals, chemoattractants secreted by oocytes form gradient and sperm direct swim to the source by altering flagella beating [13,54]. In sea urchin *Arbacia punctulata*, chemoattractant resact (a 14-mer peptide) binds to a receptor-type guanylyl cyclase and triggers the signaling events and increase the Ca^2+^ concentration in flagellar membrane [70,71,88]. Other than resact, amino acid derivative, fatty acids, and steroids are known to regulate sperm motility [73,74,75].

A chemotaxis-like mechanism is also reported in *C. elegans*, where PGs provide cues to the sperm to move towards the spermatheca for fertilization and this mechanism is regulated by TGF-β [13,54,89]. The response of each spermatozoan is uneven to the chemoattractants as evident from human and mouse spermatozoan chemotaxis studies [77,90]. Further studies are required to understand the mechanism.

## 5. PG Signaling Pathways

Calcium entry into sperm cells is an important requirement for sperm motility and fertilization [47,91]. In mammals, the steroid hormone progesterone activates CatSper channels, which are exclusively expressed in spermatozoa to increase the Ca^2+^ influx in sperm flagellum [47,48]. In other animals, activation of Ca^2+^ is also implicated for sperm chemotaxis such as sea urchin [71,92,93].

PGs such as PGE1 potentiates CatSper at the low concentrations through binding sites other than that of progesterone and how the combined action of progesterone and PGs influences sperm function remains to be established [47,94,95]. In mammalian system, PGE2-EP2 signaling activates chemokines CCL-7 signaling which facilitates sperm migration to the cumulus egg complex and integrin-mediated cumulus extracellular matrix (ECM) assembly helps sperm to penetrate the oocyte [85]. Lack of PGE2-EP2 signaling resulted in chronic CCL-7 signaling and excessive expression of integrin mediated cumulus ECM assembly that makes egg resistant to sperm penetration [85,96]. There could be many yet to be characterized genes involved in PG synthesis, but mammalian genetic systems have enormous complexity at the cellular and molecular levels.

**Figure 2:**
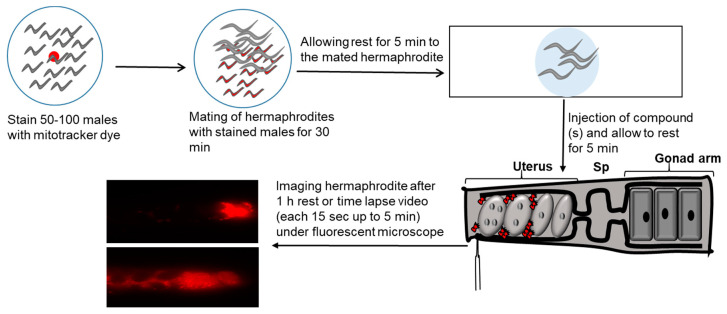
Schematic representation of method of sperm guidance assay used in *C. elegans*. Male worms incubated with MitoTracker are represented as red. Sperm guidance is evaluated by mating Mito-Tracker labeled males to adult hermaphrodites and accumulation of sperms is measured 1 h after mating in the different zones of uterus. In this assay, sperm distribution is assessed by dividing the uterus into three zones, and counting sperm in each zone. We consider a value < 70% zone 3 targeting and *p* < 0.001 as biologically significant using a t-test. Sp = spermatheca.

The signaling mechanisms by which sperms are guided to the oocyte have not been much studied due to technical challenges in the internally fertilized animals. A major challenge is the female reproductive tract’s architecture, which is inaccessible to microscopy in most species. To overcome this challenge, roundworm *C. elegans* has been used as an animal model to study the mechanism of fertilization in internally fertilized animals [97,98]. One of advantages of *C. elegans* model is the ability to screen for genes that control fundamental cell behaviors, such as sperm guidance to oocytes. In addition, transparent epidermis of *C. elegans* permits live tracking of single sperm within the reproductive tract [12,14]. Sperm guidance assay is performed by mating hermaphrodites with MitoTracker (a fluorescent dye) stained males as shown in Figure 2. MitoTracker specifically stains mitochondria. Wild type males are incubated in bacteria MitoTracker mix to fluorescently label their sperms.

We have used this assay to identify genes involved in Cox-independent PG synthesis and to investigate effects of compounds on sperm guidance in *C. elegans*.

### 5.1. Insulin/FOXO Signaling

PG synthesis in *C. elegans* is regulated by nutritional and environmental cues [12,14]. When worms sense that environment is favorable, pheromone response neurons secrete a TGF-β ligand called DAF-7 and stimulate production of Cox-independent PGs in the ovary of *C. elegans* [14]. This indicates that an environmental cue is relayed from the sensory neuron to the production mechanism of PG in the gonad.

**Figure 3:**
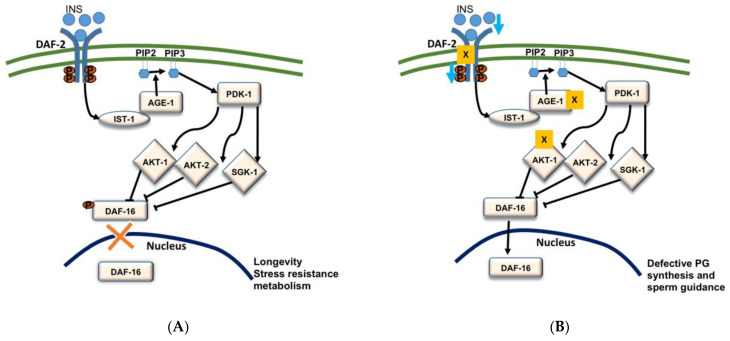
Schematic representation of insulin/insulin-like growth factor (IGF-1) signaling (IIS) pathway (**A**); Effects of IIS modification on PG synthesis and reproduction in *C. elegans* (**B**). Deletion of genes (*daf-2*, *age-1*, *akt-1*) and the reduction in insulin signaling result in increased Daf-16 activity which in turn causes defective PG synthesis and sperm guidance [12].

Abnormalities in endocrine signals such as insulin are associated with reproductive output in obese individuals [99]. We previously reported that insulin/FOXO pathway regulates PG synthesis in *C. elegans* [12]. Insulin is an evolutionarily conserved protein hormone associated with glucose metabolism increasing reproductive capacity. Among more than forty encoded insulin type peptides, *C. elegans* has only one insulin type receptor [100,101]. The phosphoinositide-3-phosphate (PIP3) mediated phosphorylation cascade get stimulated by activation of phosphoinositide-3 kinase AGE-1 by ligand binding to DAF-2 insulin receptor. Activation of PIP3 results in activation of PDK-1 kinases following activation of AKT-1, AKT-2, and SGK-2 kinases, which phosphorylate the Forkhead Box O transcription factor DAF-16 [12,101,102]. The phosphorylation of a transcription factor FOXO/DAF-16 inhibits its entry to the nucleus (Figure 3A). In *C. elegans*, loss/suppression of FOXO/DAF-16 results in small brood size and late progeny production [103,104,105].

In *C. elegans*, *daf-2*, *age-1*, and *akt-1* deficient mutants show sperm guidance defects and these effects are suppressed by the loss of *daf-16* [12]. Previous studies have indicated that any tense conditions that cause to block IIS pathway would increase the transcriptional activity of DAF-16 by inducing the translocation of DAF-16 to the nucleus (Figure 3B) [106]. This suggests that the deletion of *daf-2*, *age-1*, and *akt-1* increases the transcriptional activity of DAF-16/FOXO and its translocation to the nucleus. The mass spectrometry studies by Edmonds et al. (2010) in *daf-2(e1370); daf-16(mu86)* mutants indicated that the increased DAF-16 activity in *daf-2* mutants causes low levels of RME-2 dependent PGs which have resulted in small brood size and late progeny [12]. Edmonds et al. (2010) also demonstrated that continuous insulin signaling supports the sperm guidance [12]. In the case of insulin loss, DAF-16 that acts in the intestine, enters into the nucleus, and represses the vitellogenin expression in the intestine resulting in down-regulation of the yolk synthesis and transportation of PUFAs to the oocytes [12,107]. Vitellogenins (the principal yolk lipoproteins) carry and transfer PUFAs to the oocytes in the form of yolk-lipoprotein complex which after endocytosis by the oocytes are converted to PGs by an unknown enzymatic mechanism [12,45]. Overexpression of DAF-16B in germ line, compromises the endocytosis of yolk lipoprotein which results in the severe sperm guidance defects [12]. In a nutshell, these results indicate that insulin signaling promotes yolk transport to the oocyte and facilitates the PG synthesis in *C. elegans*.

### 5.2. DAF-7/TGF β Signaling

**Figure 4:**
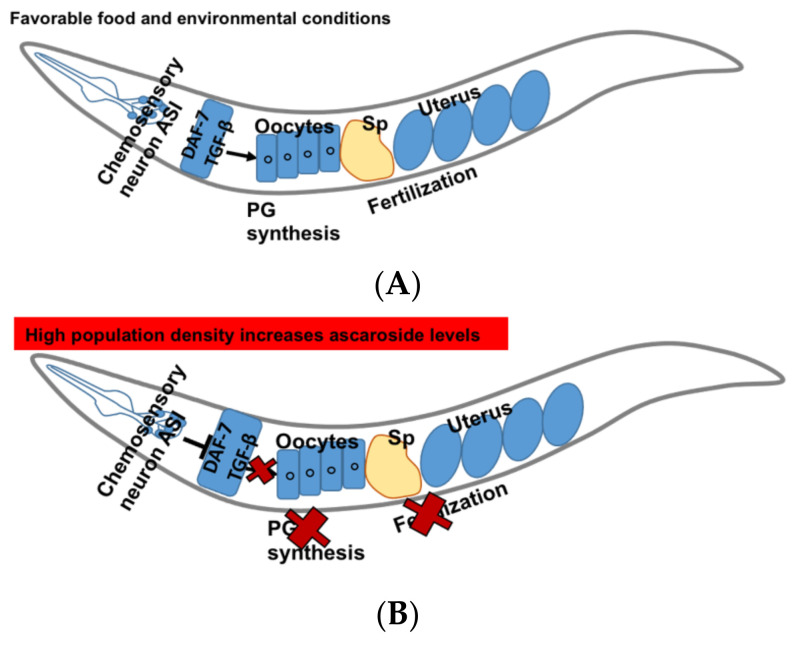
DAF-7/TGF-β signaling pathway in *C. elegans*. (**A**) Function of DAF-7/TGF-β in favorable condition. (**B**) DAF-7/TGF-β signaling in response to high population density results in increase in ascarosides levels, which in turn inhibits PG synthesis and fertilization events in *C. elegans*.

DAF-7 secreted by ASI neurons is a ligand of TGF β pathway in *C. elegans*. Various environmental cues such as pheromones regulate its secretion. In favorable conditions, amphid single (ASI) sensory neurons send the signals to the oocytes and the oocytes synthesize sperm guiding PGs (Figure 4A) [14]. In *C. elegans*, PG synthesis and sperms guidance are regulated by the pheromones “ascarosides”. The high levels of dauer pheromones such as ascarosides asc-C6-MK, asc-∆C9 represent the population density [108]. These ascarosides show concentration dependent activities. At lower concentrations (femto-lower nanomolar), they show male-attractant activities whereas at higher concentrations reproduction is down-regulated to avoid overcrowding [109].

The expression of DAF-7 is down-regulated by increased levels of ascarosides which inhibit the PG synthesis in the oocytes and cause sperm guidance defects (Figure 4B). DAF-7 transduces signals to DAF-1 type I and DAF-4 type II receptors, and downstream R-Smads DAF-8 and DAF-14 act to inhibit the Co-Smad DAF-3 [110,111,112]. It has been shown that DAF-7 regulates fat metabolism and feeding behavior and deletion of *daf-7* results in the accumulation of fat in the intestine [113].

Mutation in *daf-7*, the *daf-1* and *daf-4* receptors, or downstream *daf-8* and *daf-14 R*-Smads results in sperm guidance defects and the defects can be rescued by the loss of antagonistic co-Smad *daf-3* [14]. Recently, we have shown that reduced levels of PGs cause sperm guidance defects and suppression of *daf-3* rescues the PG production as well as the sperm guidance defects in *daf-1* mutants [89]. RNA sequencing results of WT, *daf-1* and *daf-1:daf-3* established the differential regulation of neuronal neurotransmitter transporters and ion channels genes [114]. Interestingly, feeding of PG precursor (i.e., AA) to *daf-1* mutants failed to rescue the sperm guidance defects and PG synthesis. However, an in vitro chemical reaction between *daf-1* lysate generated by lysing worm pellets and AA resulted in the production of PGF2α indicating that *daf-1* mutants have machinery required for PG synthesis [89]. The reason for sperm guidance defect and low levels of PG in *daf-1* mutants could be due in part to the inaccessibility of AA. These results also suggest that DAF-7 works non-autonomously to regulate important neurocrine factors that may affect the transportability and release of AA to the oocytes.

The DAF-7/TGF- β pathway is associated with PG production, based on the fact that *daf-1* Type I receptor mutants show low levels of F2-series PGs in *C. elegans*. The decline in PG levels causes sperm guidance defects in *daf-1* mutants. Thus, this mechanism is a key pathway connecting environmental conditions to reproductive fecundity.

## 6. Conclusions and Future Directions

It has been established that PGs can be produced by different pathways and are involved in the female reproduction. In response to specific environments, Cox-independent PGs are formed through a novel, biologically regulated enzymatic mechanism where they regulate sperm motility and fecundity in *C. elegans*. Identification of this unconventional PG synthesis opens up the possibility that altering the metabolic pathways, either through genetic mutation, dietary changes, or environmental exposures could modulate fertility and thus reproductive health.

Since omega-3 and omega-6 PUFAs are precursors of PGs, they may have a significant impact on insulin signaling that, in turn triggers PG synthesis and reproduction. Although there has been significant progress in PG research and understanding PGs roles in fertilization, a number of questions still remain unanswered. Particularly, key enzyme(s) other than Cox associated with conversion of AA to PGs and inhibitors that selectively inhibit Cox-independent PG synthesis are still unknown and warrant further investigation.
